# Does an Imitation Strategy Promote Long-Term Firm Growth in a Dynamic Environment? A Meta-Analysis

**DOI:** 10.3389/fpsyg.2021.774071

**Published:** 2021-12-09

**Authors:** Huatao Peng, Chen Zhou, Bert M. Sadowski, Tingshu Sun

**Affiliations:** ^1^School of Management, Wuhan University of Technology, Wuhan, China; ^2^School of Innovation Sciences, Eindhoven University of Technology, Eindhoven, Netherlands; ^3^School of Entrepreneurship, Wuhan University of Technology, Wuhan, China

**Keywords:** imitation strategy, firm growth, long term, dynamic environment, meta-analysis

## Abstract

The increasing number of successful latecomer enterprises has led to a growing research interest in the area, but there is a lack of consensus in academic circles on the relationship between imitation strategy and firm growth. While some enterprises achieved sustainable growth based on an imitation strategy, others withdrew from the market soon after their initial market entry. In this context, this meta-analysis synthesizes empirical findings including 23 independent samples (*N* = 66,110) to obtain evidence and explore the extent to which an imitation strategy affects firm growth. Moreover, by further examining the moderating effects of industry conditions, country-specific factors, and performance time horizons, this research also aims to address a complementary research question: in which context is imitation strategy more beneficial for the firm growth? We found that an imitation strategy is more effective in promoting firm growth in low-tech industries than in high-tech industries and in non-OECD countries than in OECD countries. It fosters the short-term performance rather than the long-term performance of a firm. Our research findings are meaningful for enterprises to choose an appropriate imitation strategy according to their unique attributes, enabling sustainable growth in a dynamic environment.

## Introduction

Recently, a large number of latecomer firms, such as Lyft (the follower of Uber, the ride-hailing service enterprise) or Hellobike and Mobike (followers of Ofo, the bicycle sharing enterprise), have been able to maintain their market position even in the face of fierce market competition in rather dynamic environments. The increasing number of successful latecomer firms has attracted great attention in the academic and managerial community. However, scholarly knowledge has been limited with respect to the extent to which an imitation strategy can promote the sustainability of firm growth in dynamic environments. On the one hand, the imitation strategy is likely to boost firm growth (Fu and Tietz, 2019). For example, a study by Golder and Tellis (1993) involving 50 different categories of products showed that pioneering firms had an average market share of only 10% and a failure rate of 47% over the long term. In contrast, latecomer firms had a lower failure rate (8%) and a larger average market share (28%). Fu and Tietz (2019) further observed that imitative latecomer firms tended to have easier access to investment since these latecomers adopted an imitation strategy that faces less market and technological uncertainty compared to market pioneers. In addition, an imitation strategy often allows firms to better attract consumers and obtain considerable economic benefits because they can produce products at lower costs and sell them at lower prices (Sajeva, 2013). On the other hand, there is also some anecdotical evidence indicating that enterprises applying an imitation strategy are less likely to increase profitability because first movers already gained brand loyalty and were able to influence the preferences of customers (Schmalensee et al., 1982; Frynas et al., 2006). Additionally, some research revealed that an imitation strategy may reduce innovation gains leading to a negative impact on firm performance (Zheng Zhou, 2006; Bouncken et al., 2015; Xia and Liu, 2017). Meanwhile, an imitation strategy seems hard to sustain while fostering the survival and competitiveness of an enterprise (Moon and Acquaah, 2020). In general, there has hardly been any consensus in academic circles on the extent to which an imitation strategy might promote firm growth over the long term in a dynamic environment.

The mixed results of these studies might be due to the fact that diverse attributes have been investigated in the different studies. However, these attributes seem to have a rather different impact and might cause high variability and uncertainty in cases when an imitation strategy is implemented (Baradello and Salazzaro, 2012; Posen et al., 2013; Benhabib et al., 2014). In this context, the study aims to investigate the extent to which the imitation strategy–firm growth relationship is influenced by other factors accounting for these diverse attributes in the selected samples.

Organizational learning theory reveals the dynamic and phased characteristics of the firm learning process (Corbett, 2005; Man, 2006). As a form of organizational learning (Yang and Hyland, 2006), imitation also needs to be examined within the external dynamic environment to account for adaptation of the firm during the different growth stages (Teece et al., 1997). These external factors influencing the implementation process of an imitation strategy are industry conditions (Perla and Tonetti, 2014; Buera and Oberfield, 2020; Baslandze et al., 2021), the specifics of the countries under investigation (Williamson and Cable, 2003; Zheng Zhou, 2006; Lee and Zhou, 2012), and the time horizon of firm performance (Kim and Nelson, 2000; Moon and Acquaah, 2020). However, little work has been done to study the effectiveness of an imitation strategy by focusing on these factors. Previous studies usually have used selected samples in very specific areas, so more general conclusions could hardly be drawn.

To fill the above research gaps, the objective of this research is to contribute several new insights to the literature. First, the authors of this paper aim to identify more general patterns in the relationship between imitation strategy and firm growth by providing a quantitative empirical aggregation of prior empirical studies regarding imitation strategy and firm growth. A single empirical study might face sampling, measurement, stochastic, and external validity problems. However, a comprehensive and systematic analysis of the imitation strategy–firm growth relationship, to our best knowledge, has not been undertaken. Meta-analysis has a rigorous and systematic calculation procedure to aggerate prior empirical studies, so it can overcome limitations in a single empirical study (Raudenbush et al., 2004). The main effect test during the meta-analysis process enables us to realize the quantitative correlation analysis of the relationship and examine the universal relationship between imitation strategy and firm growth. Second, this study attempts to examine whether there are inconsistencies in the aggregated quantitative research results through the homogeneity test during the meta-analysis process. If these inconsistencies in prior empirical studies exist, this study intends to generate a reasonable explanation for the apparent inconsistencies of the research results through the moderating effect test during the meta-analysis (Raudenbush et al., 2004). Specifically, this study would further test whether these inconsistencies are influenced by some factors such as industry conditions, country-specific factors, or performance time horizons that might cause these inconsistent research results and the extent to which they might act as moderating factors. Third, this study seeks to develop a framework to characterize the mechanisms under which imitation strategies affect firm growth in a dynamic environment. Specifically, this study would provide the systematic construction of a framework studying the link between an imitation strategy of a firm and its long-term growth under varying industry conditions and in different countries. Fourth, the findings of this study can provide new insights for policymakers to promote long-term firm growth.

The remainder of this paper is organized as follows: In section “Theoretical Framework and Hypothesis Development”, the theoretical framework derived from the literature is constructed, and different hypotheses are developed. In section “Method and Data”, the different samples, data, and research methods are illustrated, followed by the description of the findings in section “Findings”. In section “Discussion”, the paper discusses the key findings, limitations, and implications for future research. Finally, the conclusions are presented in section “Conclusion”.

## Theoretical Framework and Hypothesis Development

### Imitation Strategy and Sustainable Firm Growth

Within their respective traditions, resource-based theories of the firm, neo-institutional approaches, and theories on organizational learning have extensively been used in the past to analyze the relationship between the imitation strategy and firm growth. From the perspective of the resource-based theory of the firm, firm-internal resources and assets are the basis for an enterprise to survive. If an enterprise adopts appropriate development strategies to make full use of their valuable and rare resources and assets, it will be able to improve its long-term performance (Duncan, 1972; Fernhaber and Li, 2010). For example, through an imitation strategy, an enterprise can fully integrate and make use of firm-external resources, invoke its firm-internal experiences and knowledge, and continuously improve its absorptive capacity, thereby achieving and maintaining its competitive advantages (Wu et al., 2019). Based on neo-institutional theory, enterprises have to manage their development in uncertain contexts and capital markets (Mcgrath and Macmillan, 1995). The uncertainty in these environments prompts enterprises with inferior market positions to adopt an imitation strategy. Due to a high level of uncertainty in dynamic environments based on rapid technological change and intense market competition, first-mover enterprises might have the highest quality of market information and the most advanced technology. However, enterprises imitating the first mover in the market can achieve better market performance as these uncertainties resolve (Haveman, 1993; Semadeni and Anderson, 2010).

From the perspective of organizational learning, an imitation strategy is a type of organizational learning used by firms based on observation and learning leading to similar actions (Yang and Hyland, 2006). With an imitation strategy, enterprises can develop certain capabilities by using learning that are new to the organization but are not necessarily new to the market (Shenkar, 2010a). Research also shows that an imitation strategy can allow enterprises to quickly understand and learn the features and technologies underlying a new product launched by a first mover, thereby reducing or avoiding high development and testing costs and increasing the probability of success, resulting in a superior performance (Lieberman and Asaba, 2006; Lee and Zhou, 2012; Doha et al., 2018). The promising benefits of an imitation strategy in a dynamic environment has recently received widespread attention in the literature (Collins, 2015; Cerqueti et al., 2016; Bi et al., 2017; Doha et al., 2018). Imitation strategies by enterprises may not only gain from continuous feedback from the market and from higher profitability not only by providing similar products at a lower price (Shenkar, 2010a) but also by actively generating a sustainable competitive advantage to the firm over the long term (Lee and Tang, 2018; Ali, 2021).

However, empirical studies have shown that the results of some of these predictions are rather mixed. Some studies have indicated that an innovative strategy can lead to more technological strengths and size-related flexibility leading to a higher firm performance (Giachetti and Torrisi, 2018; Ali, 2021). In addition, research has shown that an imitation strategy may not guarantee the short-term survival as well as long-term competitiveness of an enterprise (Moon and Acquaah, 2020). Considering that in a dynamic environment, the competitive advantage for a firm using an imitation strategy is of rather limited value, enterprises still have to become more innovative to gain marketing advantages and create value over the long term.

Although research has also shown that an imitation strategy may negatively influence the innovative performance of a firm, a large number of latecomer enterprises catch up with first movers in the market by implementing an imitation strategy as demonstrated by enterprises like Lyft or Hellobike. In dynamic environments, firms facing high uncertainty may use an imitation strategy to gain from advertising spillovers from the first movers of the market, enjoy the new markets pioneered by these companies, and reduce their own market development costs and marketing expenses. At the same time, an imitation strategy can utilize the knowledge spillovers by the first mover, reduce the uncertainty surrounding the technology choice, and minimize the “trial and error” cost of business management through low-cost imitation and learning. Therefore, the imitation strategy will effectively allow to reduce the business risks of the enterprise, and consequently, achieve greater efficiency with respect to technological innovation and performance improvement.

From the theoretical discussion, we derived the following hypotheses:

**Hypothesis 1.** An imitation strategy has a positive impact on firm growth.

Organizational learning theory emphasizes the dynamic and phased nature of the learning process of a firm (Corbett, 2005; Man, 2006). Enterprises not only need to optimize dynamic capabilities to adapt to different stages of firm growth (Teece et al., 1997), but must also constantly adjust their business strategies according to the external environment (Teece, 2000, 2012; Owusu and Habiyakare, 2011). As a form of organizational learning (Yang and Hyland, 2006; Ali, 2021), the characteristics of an imitation strategy are related to the dynamic environment and the growth phases of the enterprise. As the commodity markets mature and consumer demand changes, enterprises can actively adopt an imitation strategy in case they want to enter a market rather quickly (Mansfield et al., 1981; Niosi, 2012). In mature commodity markets, consumers may have a higher demand for quality and innovative attributes of commodities (Valdani and Arbore, 2007; Dobson and Safarian, 2008; Sajeva, 2013). At a later stage of market growth, enterprises may need to adjust their business strategy to adapt to these dynamic changes in consumer demand. Furthermore, the time horizon may affect the implementation process of a business strategy, which means that the enterprise may need to adopt its business strategy according to the goals at different stages of market growth to achieve superior long-term performance. In addition, the external environment may also affect the process of the implementation of an imitation strategy (Zheng Zhou, 2006; Efendi et al., 2020). By extension, in the country-specific context in terms of economic development, the legislative and policy environment matters and may restrict the implementation of an imitation strategy of a company (Mansfield et al., 1981). Finally, the high requirements for novelty and creativity in a knowledge-intensive enterprise also seem to hinder the implementation of an imitation strategy (Dobson and Safarian, 2008; Sajeva, 2013). Therefore, in addition to the analysis of the relationship between imitation strategy and firm growth, this study also includes industry conditions and country-specific factors as moderating factors. The objective of this comparison is to investigate the relationship between imitation strategy and firm growth under different industry conditions in a variety of countries with respect to company performance to gain new insights about the long-term effects of this strategy.

### Moderating the Effect of Industry Conditions on the Imitation Strategy–Firm Growth Relation

Within the external environment of enterprises, industry conditions exercise an important influence on the relationship between imitation strategy and firm growth. The industry conditions of enterprises are heterogeneous, and the technological opportunities and interaction patterns in different industries are also different. While high-tech sectors emphasize research and development (R&D), low-tech sectors tend to rely on technology acquisition. Therefore, enterprises need to choose effective development strategies to adapt to specific industry conditions (Conte and Vivarelli, 2014). Compared with a low-tech industry environment, implementing an imitation strategy in a high-tech industry environment is likely to be more problematic and lead to lower profitability over time. As high-tech enterprises are typically very technology-intensive with a high demand for continuous innovation, these companies rely on high consumer demand after market entry which allows to develop the new market rather quickly. As a result, high-tech enterprises can charge a premium price and gain considerable economic benefits shortly after they enter the market (Baradello and Salazzaro, 2012), which is essential to sustain profitability over the short term and promotes corporate growth in the long term. In these industries, intellectual property, including knowledge, trade secrets, and know-how remains vital for the growth of these enterprises (Dollinger, 2008). To maintain these advantages over the long term, patents and product designs must be strictly protected by intellectual property law (Franzoni and Kaushik, 2016). As a result, latecomer companies in high-tech industries face difficulties in entering the market based on an imitation strategy. Furthermore, an imitation strategy may not be efficient and effective in an high-tech industry as this sector is characterized by a higher level of uncertainty and rapid technological change (Amabile, 1997; Baradello and Salazzaro, 2012). In contrast, an imitation strategy is likely to be more successful in a low-tech industry. This is because the growth of low-tech enterprises is more dependent on gaining market share and generating economies of scale. Enterprises can often achieve these goals rather quickly and at a lower cost by using an imitation strategy (Baradello and Salazzaro, 2012; Efendi et al., 2020). Therefore, it seems less likely that an imitation strategy in a high-tech industry will lead to long term growth. The second hypothesis is as follows:

**Hypothesis 2.** As the relationship between an imitation strategy and firm growth is moderated by industry conditions, the relationship will be stronger in low-tech industries than in high-tech industries.

### Examining the Country-Specific Effects of an Imitation Strategy

In the process of implementing an imitation strategy, country-specific effects play an important role in promoting firm growth (Williamson and Cable, 2003; Zheng Zhou, 2006; Lee and Zhou, 2012). For example, the long-term growth of pharmaceutical enterprises in India has greatly benefited from copying foreign products and absorbing advanced technologies from foreign enterprises (Haley and Haley, 2012) even if these strategies have often been described as “undignified and objectionable” (Shenkar, 2010b; Lee and Zhou, 2012). Country-specific differences in implementing imitation strategies can be related to their current level of economic development. In following Haruyama and Hashimoto (2020), this level of development can be conceptualized by categorizing these countries into Southern economies and Northern economies. Both authors propose that imitation strategies are more often utilized by companies in Southern economies.

Even if Haruyama and Hashimoto (2020) categorization is rather useful to analyze imitation strategies in different parts of the world, their classification method is rather limited as it characterizes developed countries as northern economies and developing countries as southern economies. However, the dividing line between these groups of countries remains unclear and the indicators used need to be better defined. In addition, this classification method is rather static. To overcome the limitation of this classification method, it is necessary to adapt it to current development trends worldwide (Hollington et al., 2015). Organization for Economic Co-operation and Development (OECD) is an intergovernmental economic organization with 38 member countries. Compared to the rather rigid distinction between “Southern economies” and “Northern economies”, it seems more appropriate to divide the world into OECD Member countries and non-OECD countries when considering the impact of country-specific differences of an imitation strategy. First, this classification method can better distinguish between country-specific differences, because the countries need to meet specific requirements to qualify as an OECD Member country. Countries which are part of the OECD have clear regulations with respect to official development assistance (ODA). This should be not less than 0.7% of GDP. Second, this classification method is more dynamic and effective compared to other definitions. As the patterns of world development change, the number of OECD member countries has gradually expanded. When the OECD was founded in 1961, there were only 20 OECD member countries, and another 18 member countries were added successively in the following 20 years.

From the aspect of local economic development, the living standards of the general public are higher in OECD member states as the local economy in these countries is well developed. When the basic living conditions of people in countries are covered, companies also provide high-tech products of high-quality features in these markets. High-end consumer demand is rather difficult to satisfy for companies with a simple imitation strategy. Moreover, companies in OECD member countries are more likely to have sufficient economic strength to take on possible risks surrounding R&D investment. As a result, they might pay more attention to their own innovation ability rather than blindly imitate other companies. In contrast, in non-OECD countries where the economies are relatively underdeveloped, the basic needs of people are often not fully satisfied. A company will be more likely to use an imitation strategy in order to copy firms that already provide products that fill the local market demand at a low cost and sell these products at a low price. In this way, the imitation strategy can help non-OECD countries obtain feedback from the consumers in the market and promote their growth in the local market. From the aspect of sectoral belongings, the industrial structure and the pillar industries are often different in OECD member countries and non-OECD countries, and non-OECD countries tend to have more low-tech enterprises than OECD member countries (Quatraro and Vivarelli, 2015). Imitation strategy can often effectively help enterprises to achieve firm growth by obtaining market share and economic profits in less developed countries where traditional and low-tech sectors play the dominant role. Therefore, imitation strategy seems to be significant to promote firm growth in non-OECD countries than in OECD member countries. Thus, the third hypothesis can be formulated as follows:

**Hypothesis 3.** As the relationship between an imitation strategy and firm growth is moderated by country-specific factors, the relationship will be stronger for non-OECD countries than for OECD member countries.

### Long-Term Performance Effects of an Imitation Strategy

“History friendly modeling,” introduced by Malerba et al. (1999), also provide new insights for us to formally analyze the environmental dynamics related to imitation strategies adopted by heterogeneous enterprises. The external environments, including demanding conditions, technological changes, and competition from rivals should be taken into specific consideration when investigating heterogeneous enterprises since the industrial sectors are transforming and upgrading with historical evolution (Capone et al., 2019). From the aspect of an industry life cycle, the development patterns of enterprises at the sectoral level change over time (Malerba, 2002). For example, Kalpana, a United States (US) start-up company and the market pioneer in the local area network (LAN) industry, applied an incremental innovation strategy to improve the technology of the early bridge and launched the first LAN switch, which gained direct competition with bridges rather than with routers. The introduction of the LAN switch has had a profound impact on the subsequent development of the LAN industry. The LAN switch was a blow to LAN bridges. As a result, the sales of routers slowed down. In the face of a window of opportunity provided by the LAN switch to the LAN industry, a large number of new companies entered the market through imitating strategies and the fierce market competition began. With the intensification of market competition, the technological level of LAN enterprises is gradually upgrading, and customer needs are also constantly changing, and the LAN industry has thus entered a stage of diversified development. In this stage, the products launched by different enterprises in the industry are more and more similar in terms of functions, and enterprises need to provide differentiated products to gain a competitive advantage in this “demand competition” (Malerba, 2002). Therefore, it seems meaningful to adopt different phased development strategies according to industry dynamics.

One of the salient advantages of enterprises adopting an imitation strategy is that they are able to enter markets and penetrate them rapidly (Baradello and Salazzaro, 2012). For example, Dingding, a sharing bicycle enterprise in China that is one of the followers of Ofo, was not the first in launching a sharing bicycle product, but has been rather quick in entering the market through an imitation strategy. However, due to an increasing number of competitors in the market even if consumer demand was still growing, Dingding failed to survive in the Chinese sharing bicycle market. In other words, although enterprises are likely to gain short-term benefits through an imitation strategy, this strategy may be limited in providing superior firm performance in the long run (Moon and Acquaah, 2020). Furthermore, as consumers move up the “quality ladder” and no longer be satisfied with the original imitation products (Grossman and Helpman, 1991), they are opting for companies accounting for new innovative features of products and services. In particular in mature markets, consumers have a preference for high-quality products with new features (Sajeva, 2013). At this point in time, companies can no longer rely on an imitation strategy only to maintain their growth. In addition, if an enterprise intends to implement an imitation strategy over an extended period of time, the resources available for developing new products or new processes will rather be limited (Dobson and Safarian, 2008). This means that innovation capabilities of the enterprise will be rather difficult to maintain over the long time. Thus, the fourth hypothesis can be posed:

**Hypothesis 4.** As the relationship between an imitation strategy and firm growth is moderated by the time under consideration, the relationship will be stronger for the short term compared to the long term.

## Method and Data

### Method

The scientific measurement rules of meta-analysis enable scholars to explore in more detail the relationships between different variables, which is beneficial if a more complex conceptual model is tested. Due to this benefit, scholars have widely used the meta-analysis method in business management research (O’Boyle et al., 2016; Jin et al., 2017; Cravo and Piza, 2019). Combining the samples of multiple studies for statistical processing, meta-analysis can obtain results closer to reality by expanding the statistical sample size (Raudenbush et al., 2004). In this way, scholars can detect relationships in a variety of real-life situations and improve theory development. Therefore, the meta-analysis is selected in this study to explore the relationship between imitation strategy and firm growth more comprehensively over the long term.

Additionally, the meta-analysis method can also be used to test whether the results of existing research in a certain area are consistent. If the research results are inconsistent, the meta-analysis method can further test the moderating effects of the relationship between the independent variable and the dependent variables through different scientific calculation procedures to generate a reasonable explanation for the apparent inconsistencies of the research results (Raudenbush et al., 2004). Therefore, this study further examines whether there are inconsistent results in the different studies concerning imitation strategy and firm growth over the long term. In the case of any inconsistencies, this study further tests potential moderating factors such as industry conditions, country-specific factors, or performance time horizons that might cause these inconsistent research results and the extent to which they might act as moderating factors. In this way, this research will be able to provide managers with suggestions for improving their imitation strategies under certain external conditions.

The analysis was undertaken in the following way. First, we collected the existing quantitative literature with a focus on imitation strategy and firm growth from different academic databases. Second, after importing the collected quantitative research data into the meta-analysis software, we tested the publication bias of the data set derived from the literature search to ensure the validity and reliability of the data. Third, we conducted the main effect test to explore the relationship between imitation strategy and firm growth. The strength of the overall relationship between the imitation strategy and firm growth is reflected in the overall weighted average correlation coefficient (weighted $\bar{r}$). Fourth, in case the results of the quantitative research collected were heterogeneous, a further moderating test was performed. The objective of this test was to reveal the extent to which certain factors could act as moderating factors and better explain this heterogeneity.

### Collection of Studies

In the study, we consulted academic databases like EBSCO, Springer, and ScienceDirect to collect the relevant international literature in the area. At the time of retrieval, we focused on the publication time during the period 1978 and 2021. In order to include the relevant literature in the area, a combination of keywords was used to search different bibliographic databases. A keyword search in these databases was undertaken by looking for key words like “imitation” (e.g., imitation strategy, imita*, mimetic, mimic, copy*, copycat, and follower), and firm growth (e.g., firm performance, ROA, ROE, and sale growth). Then, we manually retrieved information from high-quality journals in the area of business management including Management Science, Strategic Management Journal, and the Journal of Business Venturing. In addition, we also retrieved data by searching on Google Scholar for unpublished papers in this area. In this way, we could test for publication bias and the accuracy and effectiveness of the meta-analysis results. By using these three retrieval methods, the different articles for the final dataset were collected.

To undertake the meta-analysis, we had to extract the relevant data from each collected article to further explore the relationship between the independent variable and the dependent variables. In the process of screening for these data, the study adopted the following criteria: first, the literature should focus on the theme of imitation strategy and firm growth; second, the literature should be quantitative by nature; third, the literature should also report statistical criteria such as sample size, the correlation coefficient between variables, or the value for the *t*-test.

**FIGURE 1:**
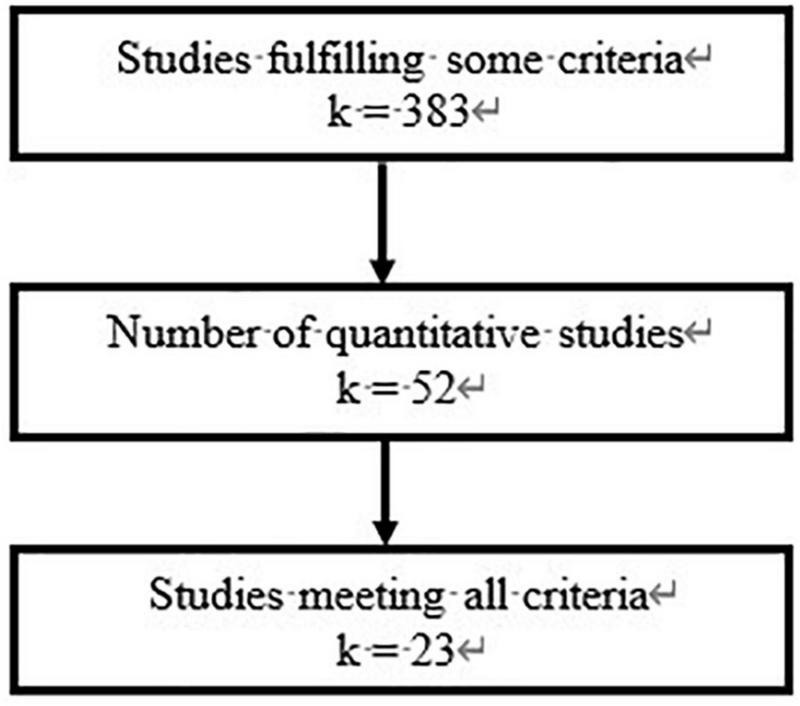
Literature screening process (*k* denotes the number of studies).

After using the above principles to screen the studies collected, we obtained 23 articles that met all requirements. The 23 articles provided 66,110 independent samples in total. The meta-analysis method requires at least 10 articles (Wilson, 1999; Murphy, 2017). Therefore, our research meets the methodological requirements. The entire literature screening process of this study is presented in Figure 1. The basic information of each selected article is displayed in Table 1.

**TABLE 1:** Basic information of the collected quantitative research.

References	Sample size	Imitation strategy	Firm growth	Investigated country	Overall correlation coefficient	Industry condition	Performance time horizon
Fernhaber and Li, 2010	150	Frequency-based imitation	Return on sales	US (OECD)	0.01	High-tech	Short-term
Sirmon et al., 2008	2531	Imitability level	Value added	France (OECD)	–0.11	Low-tech	Short-term
Ethiraj and Zhu, 2008	912	Time-based imitation	Sales growth	US (OECD)	–0.155	High-tech	Long-term
Maruta et al., 2017	180	Product imitation	Competitive advantage	Indonesia (non-OECD)	0.34	Low-tech	Short-term
Bouncken et al., 2015	169	Imitating pioneers	Innovation performance	Germany (OECD)	0.031	Low-tech	Short-term
Williamson and Cable, 2003	505	Frequency-based imitation	Return on sales	Mixed	–0.027	Low-tech	Short-term
Semadeni and Anderson, 2010	557	Imitating competitors	Offering/organization innovativeness	US (OECD)	–0.025	High-tech	Long-term
Zheng Zhou, 2006	298	Imitating products	New product performance	China (non-OECD)	0.39	Low-tech	Short-term
Wu et al., 2019	8517	Imitation strategy	Firm performance	China (non-OECD)	0.005	Low-tech	Long-term
Xia and Liu, 2017	942	Product imitation	Innovation performance	UK (OECD)	0.347	Low-tech	Long-term
Lee and Tang, 2018	147	Imitation orientation	Firm performance	China (non-OECD)	0.33	Low-tech	Short-term
Giachetti and Torrisi, 2018	347	Imitability level	Sales growth	Mixed	0.37	High-tech	Long-term
Giachetti et al., 2017	566	Imitation scope/speed	Firm performance	UK (OECD)	0.123	High-tech	Long-term
Giachetti and Dagnino, 2017	283	Imitability level	Return on sales/return on assets	Mixed	0.026	High-tech	Long-term
Lee and Zhou, 2012	192	Imitating competitors	Financial performance	China (non-OECD)	0.18	High-tech	Short-term
Soda et al., 2008	501	Imitability level	Value added	Italy (OECD)	0.06	High-tech	Long-term
Podoynitsyna et al., 2013	1431	Product/technology/ technology imitation	Return on investment	US (OECD)	0.19	High-tech	Short-term
Shu, 2012	329	Imitating competitors	Product’s market success	Mixed	0.161	High-tech	Short-term
Ali, 2021	269	Imitation strategy	Sustained competitive advantage	Korean (OECD)	0.58	Mixed	Short-term
Moon and Acquaah, 2020	486	Imitation innovation	Sales	Korean (OECD)	–0.09	Low-tech	Short-term
Frankenberger and Stam, 2020	122	Business model imitation	Firm growth	Switzerland (OECD)	0.295	High-tech	Short-term
Liao, 2019	46,476	Imitative sales	Labor productivity	Spain (OECD)	0.04	Mixed	Long-term
Efendi et al., 2020	200	Imitating capability	Competitiveness advantage	Indonesia and Malaysia (non-OECD)	0.76	Low-tech	Short-term

### Variable Coding

The collected quantitative research was standardized by using the following procedure. First, we recorded the basic information of each study, including the name of the author followed by the year of publication, the sample size of the variable names, and the country (if the investigated country belongs to OECD member countries, it would be coded as “OECD”; if not, it would be coded as “non-OECD”). Furthermore, we coded the industry condition of the investigated enterprises (high-tech or low-tech) and the length of time covered to measure the variable related to firm growth. Second, the overall correlation coefficient of each study was calculated and recorded. If a multidimensional scale for imitation strategy or firm growth was adopted in a study, the mean of the correlation coefficients would be calculated and then recorded as the overall correlation coefficient.

To improve the accuracy of the meta-analysis results, the coding process was independently performed by the three co-authors. Comparing the respective coding results finished by the three co-authors, we found that 95.65% of the initial coding results were consistent. Conflicting coding results were also resolved by agreement after the detailed discussion and negotiation by the research team. The final coding results are presented in Table 1.

#### Dependent Variable

Firm growth manifests itself as a fundamental characteristic of the gradual expansion of scale, the maturity of the company, and its stable operation (Peng and Liu, 2018). To measure firm growth, the indicators in the collected studies were mainly performance-related indicators (e.g., return on assets or return on sales).

#### Independent Variable

Imitation strategy refers to the extent to which an enterprise will grow based on observational learning and hands-on learning to replicate and develop products that are new to the organization but not necessarily new to the market (Shenkar, 2010b). The scales for measuring the imitation strategy used in this study was generally consistent with the research by Lee and Tang (2018). We mainly measured the imitation strategy with respect to the strategic tendency to follow main competitors, the willingness to enter the market, and the degree of importance attached to other competitors with imitation strategies along with the level of emphasis on imitating competitors.

#### Moderators

The research included three moderating variables, namely industry conditions, country-specific factors, and performance.

##### Industry Conditions

In order to study the effects of industry conditions on the implementation of an imitation strategy, we utilized the distinction developed by Perla and Tonetti (2014) between high-tech and low-tech industry conditions. As a result, companies operating in high-tech industries can be found in sectors like computer hardware manufacturing, software, medicine, semiconductors, telecommunications, and biotechnology. Enterprises in low tech industries are mainly concentrated in traditional manufacturing and retailing sectors.

##### Country-Specific Factors

This moderating variable refers to the country in which the analysis has been undertaken. The literature has shown that these country-specific differences are also likely to play an important role in promoting the growth of firms when they adopt an imitation strategy (Williamson and Cable, 2003; Zheng Zhou, 2006; Lee and Zhou, 2012). Taking the level of economic development of different countries into account, we divided the countries into OECD and non-OECD countries.

##### Performance

This moderating variable refers to the time-period over which the firm performance was measured. The literature has shown that an imitation strategy might have differential effects on achieving competitive superiority over the long term compared to the short term because an imitation strategy might be more challenging to maintain a long-term competitive advantage (Moon and Acquaah, 2020). Consistent with previous studies (Mathias et al., 2018; Nason and Wiklund, 2018), we chose 5 years to characterize the differences between short term and long term performance. If the period over which the performance was measured was equal to 5 years or longer than 5 years, we classified it as a long-term performance. If the performance was measured over a period less than 5 years, we classified it as short-term performance.

### Meta-Analysis Process

#### Publishing Bias Test

As meta-analysis enables a researcher to combine a variety of published and unpublished studies while allowing to conduct a comprehensive analysis of these studies. A publication bias test is required to ensure the accuracy and validity of the meta-analysis results.

Publication bias refers to the phenomenon that in similar studies, papers with positive results (studies with statistically significant results) are more likely to be accepted and published in accredited journals than papers with negative results (studies without statistically significant results) (Card, 2012). The collected data in this research include published papers in journals and unpublished studies. Therefore, a publication bias test had to be carried out to ensure the universality and representativeness of the research results. The publication bias tests are usually based on the fail-safe number (FSN). FSN represents the number of missing studies that should be added to reverse the significance of the main effect and make the main effect size not statistically significant.

Suppose that a significant *p*-value is obtained based on several studies, but a few studies may be missed. If these missed studies are included in the meta-analysis, then the *p*-value of the main effect may no longer be statistically significant. Therefore, the meta-analysis approach can calculate how many missing studies are needed to the main effect size statistically insignificant. If only a few studies are needed to reverse the significance of the main effect, then we would suspect that the actual main effect size is statistically insignificant. Conversely, if it takes a lot of research to reverse the statistical significance of the main effect, there is no reason to suspect that the true effect is insignificant. Conversely, if the FSN is larger, it would be less likely for publication bias to exist, and the meta-analysis results would be more consistent. According to the criterion proposed by Raudenbush et al. (2004), 5 × number of studies + 10 is generally selected as the criterion in the meta-analysis, that is, no significant publication bias exists only when FSN > 5 × number of studies + 10.

#### Correlational Analysis

The main effect test and moderating effect tests are shown in the correlation analysis. Through the main test, the strength of the overall relationship between imitation strategy and firm growth could be verified. Based on the suggestions of Raudenbush et al. (2004), the relationship strength would be reflected in the weighted $\bar{r}$ (the overall weighted average correlation coefficient). The result of the homogeneity test will determine whether to choose a fixed-effect model or a random-effects model for the subsequent correlation analysis. If there is a large heterogeneity between the collected studies, the random-effect model is usually used for meta-analysis. In this study, the *Q* test was used to test for statistical heterogeneity, and then *I*^2^-heterogeneity was used to quantify the results. In this way, the heterogeneity of the research results was tested.

The heterogeneity of test results also determines whether further moderating effect tests are required. If the heterogeneity test results indicate that there is considerable heterogeneity among the correlation coefficients in the collected studies, then subsequent analysis of potential moderator variables is beneficial for explaining which factors cause this heterogeneity (Higgins et al., 2003).

## Findings

### Publishing Bias Testing Results

**FIGURE 2:**
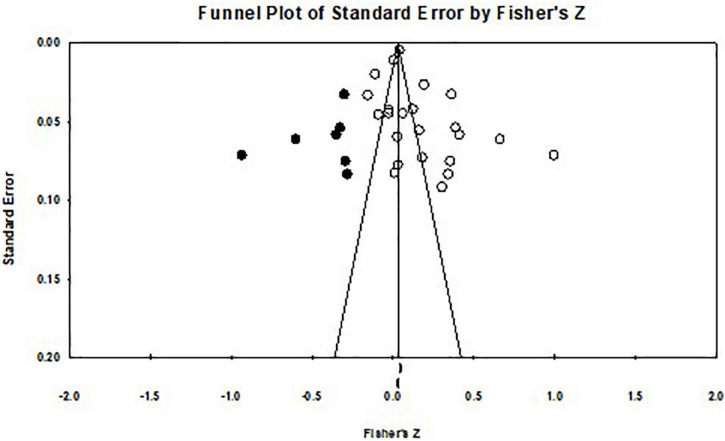
Funnel diagram of publication bias.

The publication bias test is applied to characterize the effectiveness and accuracy of the results of the meta-analysis. The publication bias test is based on the FSN, which is 1476 for this meta-analysis, larger than 5*k* + 10 (*k* donating to the number of collected studies is equal to 23 in this meta-analysis research, so 5*k* + 10 is 125). Besides, we further checked the publication bias among the collected studies by applying a funnel diagram. The results are shown in Figure 2. The funnel diagram is symmetrical and the relative data are mostly distributed at the top of the funnel plot. This indicates that there was no publication bias among the collected studies. The results of the meta-analysis result will be authentic and representative.

### Main Effect Test Results

The authors used meta-analysis software to test the main effect of the relationship between imitation strategy and firm growth. The test results are displayed in Table 2. According to the suggestion by Raudenbush et al. (2004), the strength of the overall relationship between the independent variables and the dependent variable is reflected in the overall weighted average correlation coefficient (weighted $\bar{r}$). The weighted $\bar{r}$ was calculated and obtained by applying meta-analysis software. The calculation process for the weighted $\bar{r}$ refers to the relevance of the sample size in each collected study to the total sample size, which is the sum of the sample sizes of 23 articles.

**TABLE 2:** Analysis of imitation strategy and firm growth.

Dependent variable	Research number	Total sample size	weighted $\bar{r}$	Confidence interval		Two-tailed test	
				Lower limit	Upper limit	*Z*	*p*
Firm growth	23	66,110	0.172	0.112	0.232	5.510	0.000

A total number of 66,110 articles were used to investigate the relationship between imitation strategy and firm growth. Thus, the weighted $\bar{r}$ for the main effect test was 0.172 (*p* = 0) with 0.232 as the upper limit of the 95% confidence interval and 0.112 as the lower limit. Therefore, we could conclude that the imitation strategy is positively correlated with firm growth. Thus, Hypothesis 1 was verified.

### Homogeneity Test Results

The results for the homogeneity test are displayed in Table 3. Among them, the *Q*-value was 661.729 (*p* = 0) revealing significant heterogeneity among the 23 collected research results in the different studies.

**TABLE 3:** Homogeneity test results.

Random effect model	Research number	Heterogenicity				Tau-squared			
		*Q*	Df (*Q*)	*p*	*I*-squared	Tau-squared	Standard error	Variance	Tau
Firm growth	23	661.729	22	0.000	96.675	0.020	0.016	0.000	0.14

Additionally, we found an *I*-squared value of 96.675, which showed that 96.676% of the observed variation in the relationship between imitation strategy and firm growth are caused by the variety of correlation coefficients in the collected studies. The demarcation point concerning the *I*-squared value of different heterogeneity degrees was 75/50/25. If the *I*-squared value was larger than 75%, it indicated that the correlation coefficients in the studies were characterized by high heterogeneity. By further testing for the moderating effects within the relationship between the imitation strategy and firm growth, we found that the high heterogeneity of the studies also indicated that the random effect model should be selected for correlation analysis when applying meta-analysis.

### Moderating Effect Test Results

The moderating effect test was conducted in an attempt to explain why there were inconsistent results in the collected studies. More specifically, we analyzed the moderating effects to examine whether the industry conditions, the country-specifics, and the performance measure affect the strength of the relationship between imitation strategy and firm growth. The results of the moderating tests have been as follows:

#### Moderating Effect of the Industry Conditions

The moderating effect test results for industry conditions are shown in Table 4. The imitation strategy had a significant impact on firm growth in the high-tech industry, with a weighted $\bar{r}$ of 0.111 (*p* < 0.05). The influence of an imitation strategy for firms in a low-tech industry was also significant with a weighted $\bar{r}$ of 0.217 (*p* < 0.01). The results reflected that the imitation strategy had a more statistically significant impact in a low-tech industry compared to a high-tech industry.

**TABLE 4:** Moderating effect of industry conditions.

Variables		*K*	Total sample size	Effect value	*P*-value	Confidence interval	*I*-squared
Firm growth	High-tech	11	66,110	0.111	0.025	[0.014, 0.206]	91.666
	Low-tech	10		0.217	0.003	[0.076, 0.350]	97.890

In addition, the weighted $\bar{r}$ between imitation strategy and firm growth in the high-tech industry was lower than that in low-tech industries, which meant that the relationship between imitation strategy and firm growth is stronger in a low-tech industry compared to a high-tech industry. These results verified Hypothesis 2 that the relationship between imitation strategy and firm growth is stronger in low-tech industries than in high-tech industries.

#### Moderating Effect of the Country-Specific Factors

To further explore how country-specific differences influenced the relationship between imitation strategy and firm growth, the authors tested the moderating effect of the country-specific conditions.

As shown in Table 5, in OECD countries, the imitation strategy had a significant impact on firm growth, with a weighted $\bar{r}$ of 0.363 (*p* < 0.05). In non-OECD countries, the imitation strategy was also a significant predictor of firm growth, with a weighted $\bar{r}$ of 0.102 (*p* < 0.05). These results verified Hypothesis 3 stating that the relationship between an imitation strategy and firm growth is moderated by country-specific factors, with a stronger relationship in non-OECD countries compared to OECD countries.

**TABLE 5:** Moderating effect of the country-specific factors.

Variables		*K*	Total sample size	Effect value	*P*-value	Confidence interval	*I*-squared
Firm growth	Non-OECD	6	66,110	0.363	0.014	[0.078, 0.593]	98.107
	OECD	13		0.102	0.017	[0.018, 0.185]	96.559

#### Moderating Effect of Performance Over Time

To further explore how the performance dimension influences the relationship between imitation strategy and firm growth, we tested the moderating effect of performance over time.

As shown in Table 6, in the long term, the imitation strategy had a significant impact on firm growth, with a weighted $\bar{r}$ of 0.087 (*p* < 0.01). In the short term, the imitation strategy also had a significant impact on firm growth, with a weighted $\bar{r}$ of 0.237 (*p* < 0.01). These results verified Hypothesis 4, which argued that the relationship between an imitation strategy and firm growth is moderated by the time period over which performance is measured. This relationship is stronger for companies over the shorter term rather than in the longer term.

**TABLE 6:** Moderating effect of the performance time horizons.

Variables		*K*	Total sample size	Effect value	*P*-value	Confidence interval	*I*-squared
Firm growth	Long-term	9	66,110	0.087	0.009	[0.021, 0.151]	95.813
	Short-term	14		0.237	0.002	[0.092, 0.373]	97.112

## Discussion

### Highlights of Key Findings

In line with previous research, the paper demonstrated that an imitation strategy has a positive impact on firm growth (see Hypothesis 1). This conclusion is consistent with the research results by Zheng Zhou (2006), Efendi et al. (2020), Frankenberger and Stam (2020), and Ali (2021). A firm will not only make full use of internal resources to carry out production activities but will also fully draw on external resources to provide support for their production activities, thereby enhancing its competitive advantages and promote product upgrading, and service enhancement and processes optimization. In this way, the firm can maintain an advanced position under conditions of fierce market competition while realizing firm growth. From the perspective of neo-institutional and learning theories, an imitation strategy is a choice for enterprises when they have to adapt to rapidly changing environments, and it also enables firms to benefit from learning behavior aimed at improving market competitiveness (DiMaggio and Powell, 1983; Abrahamson and Rosenkopf, 1993; Wu et al., 2019). As first, mover companies are able to obtain high-quality market information, a comfortable competitive position in the market and advanced levels of technology, imitating enterprises can follow the strategies of these enterprises by observing and learning from these firms, which reducing, in turn, market uncertainty (Semadeni and Anderson, 2010; Giachetti and Torrisi, 2018). As a result, a large number of firms prefers to apply an imitation strategy although they may sacrifice novelty for less market and technology uncertainty (Fu and Tietz, 2019). This result is contrary to the results of studies indicating that an imitation strategy may hinder the sustainable growth of firms over the long term in a highly competitive market as enterprises need novelty to stay ahead of competitors in these markets (Semadeni and Anderson, 2010; Moon and Acquaah, 2020). Suarez et al. (2015) posit that it is not worthwhile for latecomer firms to adopt an imitation strategy, because the company acting as a market pioneer has already occupied a dominant position in the market, resulting in less options to gain substantial profits by imitating the pioneering enterprise. However, for new entrant firms, it may be not essential to keep certain market positions to facilitate firm growth. Instead, the ability to learn and absorb through an imitation strategy might be more effective for the development of the enterprise over the long term.

We were able to show that the relationship between an imitation strategy and firm growth is moderated by industry conditions. This relationship will be stronger in low-tech industries compared to high-tech industries (see Hypothesis 2). From the perspective of the contingency theory, this result echoes the view by Hall et al. (1968), proposing that firm growth is greatly influenced by other conditions such as the industry environment and organizational processes within the firm. Meanwhile, this result is also consistent with research by Perla and Tonetti (2014), König et al. (2016), and Buera and Oberfield (2020) stating that the technical complexity of a particular industry will affect the implementation process of an imitation strategy. A further new insight put forward by Baslandze et al. (2021), which is in line with this result, refers to the heterogeneous effects of knowledge spillovers on firms with an imitation strategy in industries with different technological complexity. In high tech industries, there are also a large number of tech enterprises that have greatly facilitated their growth based an imitation strategy (Shenkar, 2010a; Gnyawali and Park, 2011; Moon and Acquaah, 2020). For some companies, the research results seem at a first glance rather counterintuitive. For instance, the success of Huawei, a world-renowned Chinese smartphone enterprise, is a typical example of a successful imitator. In the early 21st century, Huawei heavily relied on imitating global telecom equipment producers to quickly enter the telecommunications equipment market. Thanks to the imitation strategy, Huawei attracted a growing number of local Chinese consumers with its low-cost mobile phones and quickly conquered the rural and small-town market in China. One possible explanation for the counterintuitive result of this research is that although the imitation strategy for high-tech enterprises can indeed promote enterprises to enter the market rather quickly with lower costs and lower risks in the early stages of development, the imitation strategy will no longer guarantee the development of enterprises at a higher level as enterprises continue to climb in the high-tech value chain. Therefore, an imitation strategy seems to have just limited effects on the development of high-tech enterprises. This might be the reason why Huawei could no longer simply rely on an imitation strategy in its later development stage but had to shift its focus to become an innovation leader. With maturing technology and market growth in the high-end segments, Huawei has continuously increased R&D investment during company growth and actively innovated based on gaining from customer experience. As a result, Huawei was able to grow, not only successfully surpassing Ericsson to become the largest telecommunications equipment enterprise of the world, but even surpassing Apple to become the second-largest smartphone manufacturer of the world after Samsung (Baslandze et al., 2021). On the contrary, enterprises in industries with low-tech complexity tend not to pursue technological innovation urgently. Instead, they are under pressure to enter the market in a short period of time and rapidly gain market share. Imitation strategies are more successful for companies operating in low-tech industries. Another possible explanation that the relationship between imitation strategy and firm growth is stronger in low-tech sectors than in high-tech sectors is that low-tech sectors are generally populated by smaller and younger firms that are focusing more on incremental innovation rather than on R&D-based radical innovation. Smaller and younger firms are generally characterized by a low degree of liquidity and a high degree of diversification, which make it hard for them to get access to external financial funds and deal with the uncertainty of technology R&D. Besides, unlike large firms, the young and small firms are often unable to rely on scale and scope economies to embark on R&D projects (Ortega-Argilés et al., 2009; Conte and Vivarelli, 2014). Therefore, limited internal resources and insufficient external support restrict the smaller and younger firms to achieve firm growth through a radical innovation strategy. Instead, an imitation strategy can help smaller and younger firms break through the bottleneck of insufficient internal and external resources, effectively reducing the uncertainty and investment cost in the process of changing the fundamental principles and ideas of existing technologies. In this way, firms can imitate and constantly update existing technologies to achieve incremental firm growth. In contrast, high-tech industries are generally populated by larger and older firms, and these enterprises can promote firm growth through radical innovation based on abundant internal and external resources. Sufficient R&D resources enable these firms to acquire knowledge in a knowledge-intensive industrial environment and improve their ability to recognize, absorb, and apply knowledge. In this way, innovation premiums could be created by these larger and older firms to obtain more sustained competitive advantages. Therefore, imitation seems to be more successful in low-tech industries than in high-tech industries.

To a certain extent, this conclusion can enlighten macro-level innovation policymakers. The commercial innovation encouragement policy for small and young enterprises in the low-tech industry needs to be designed and enforced to support these enterprises in technological R&D. These small and young firms are often unable to embark on innovation activities because of insufficient R&D resources. As a result, they sometimes have to adopt imitation strategies to deal with the temporary thorny business survival issues. However, innovation is the driving force for the long-term development of an enterprise, and R&D expenditure is the main engine that guarantees innovation capability improvement and firm growth (García-Quevedo et al., 2014). The European Union (EU) has promulgated a series of policies to support small and young business innovation to reverse the continuing innovation gap between European countries and the US. Before the policy was promulgated, the industrial structure of EU was still dominated by traditional, low and medium-tech sectors. There were a large number of young and small enterprises, but their innovation capabilities were generally low, and the early failure rate for them was generally high. However, similar kinds of firms in the United States not only have a higher survival rate but also often can develop new products in their core business areas. For this reason, the EU has begun to promulgate and implement some encouragement policies to support the development of small and young enterprises, such as facilitating their access to funding to support the commercialization of innovations (Pellegrino and Piva, 2020). From the aspect of sectoral belonging, small and young enterprises in low-tech sectors are hard to achieve the innovative advantages by their own (Ortega-Argilés et al., 2009). Therefore, R&D policy addressed to these firms is likely to be surprisingly effective and beneficial. To provide better support for innovation activities of small and young enterprises in low-tech sectors, several key points should be considered when designing the R&D policy. First, policymakers should focus on providing incentives for long-term investment and commitment to R&D activities of small and young enterprises in low-tech sectors. Specifically, access to tax credits or R&D subsidies should be facilitated for these firms (Añón-Higón et al., 2015). Second, policymakers need to guide these enterprises to improve the internal development in terms of human resources, production technology, and organizational innovation. Third, policymakers should provide sufficient support for small and young enterprises in the low-tech industry to obtain diversified R&D investment. Specifically, policymakers could enforce cooperative encouragement policies to promote in-depth cooperation among enterprises, universities, and research institutions and the formation of a knowledge spillover effect (Ortega-Argilés et al., 2009).

The study also showed that the relationship between an imitation strategy and firm growth is moderated by country-specific factors as the relationship will be stronger for non-OECD countries compared to OECD countries (see Hypothesis 3). This result further supports the view by Haruyama and Hashimoto (2020) arguing that, in less developed economies, companies should focus more on imitative production of “old” products. This result may also explain why some Indian pharmaceutical firms tend to thrive on copycat strategies. The economic development of a country not only affects the demand level in the consumer market but is also embedded in the legal system and the level of technology in the particular country. These factors may also affect the implementation of imitation strategies by local enterprises. From the aspect of technological advancement, countries with advanced technological levels also provide advantages for firms with R&D capabilities. Factors such as the flexibility of the labor market, technological level, and commercial infrastructure in the business environment have severely hindered enterprises from developing R&D activities in less developed countries (Aghaei and Sokhanvar, 2020). Therefore, the development for enterprises in non-OECD countries is generally based on imitation and technological transfer, rather than on radical innovation heavily relying on domestic R&D investments. From the aspect of the local legal system, the degree to which the local patent laws restrict production processes will greatly affect the space of imitation available for an enterprise (Haley and Haley, 2012). For instance, before India signed agreements on the Trade-Related Aspects of Intellectual Property Rights (TRIPS) in 1995, India had not yet started to implement strict patent protection laws with Indian pharmaceutical companies experiencing high growth by utilizing R&D based on reverse engineering (Kale and Little, 2007).

However, the results for the moderating effects of the country-specific factors seem contrary to the conclusions of some recent studies. For example, the research by Zhan et al. (2020) shows that China, a non-OECD country, is striving to transform from an imitator to a top global innovator. Encouraged by macroeconomic policies, some Chinese enterprises no longer rely on imitation but have strengthened their R&D capabilities. This strategic adjustment has promoted the growth of some Chinese enterprises and narrowed the gap between them and their counterparts in developed countries. One possible explanation for this is that the economic patterns of world development are dynamic and fluctuating. With the advancement of the technological revolution worldwide, skill-biased technological changes transfer across international borders (Berman and Machin, 2000). To a certain extent, the early development trend of developed countries might predict the later development trend of developing countries. Just as the US industrial skills upgrading trend in the 1980s was a good predictor for the industrial skills upgrading trend in less developed countries during the 1980s (Berman and Machin, 2000). Foreign direct investment (FDI) might also lead to a greater demand for high-level technological products and skilled workers in less developed countries (Feenstra and Hanson, 1997). Therefore, although some countries are not yet OECD countries, their economic strength is constantly approaching the level of OECD member countries. Instead of completely relying on imitation strategies, enterprises in non-OECD countries should pay more attention to the R&D capabilities in the later development. Another possible explanation is that the growth within non-OECD countries has been rather uneven, with gaps emerging between rich and poor geographical areas within a country. There may be some enterprises in developed areas, in particular cities, that need to readjust their imitation strategies while others located in poorer areas can still rely on imitation strategies to promote their development. Policymakers need to design differentiated policies according to different national conditions (Fagerberg and Godinho, 2004) and pay more attention to enterprises that heavily rely on imitation strategies in non-OECD countries. Enterprises in non-OECD countries should have convenient access to public financial assistance, otherwise, these enterprises will be ignored by conservative and short-term-oriented capital markets. Specifically, policymakers can provide these enterprises with micro-credit support. In this way, these firms can also have additional R&D resources to improve the innovation capacity which is essential for their long-term growth.

We showed that the relationship between an imitation strategy and firm growth is moderated by variance in the firm performance over time (see Hypothesis 4). This relationship has been stronger over the shorter term rather than over the longer term. These results partly support the view by Kim and Nelson (2000) stating that an imitation strategy is relatively difficult to maintain over the long term. One possible interpretation for this result is that the R&D capabilities of the enterprise are gradually improving with the development of the enterprise. In order to achieve short-term strategic goals, enterprises can rarely accumulate sufficient innovation capabilities. In this situation, it is rather difficult for enterprises to rely on independent innovation to improve their performance in the short term, and the imitation strategy seems better suited to promote company growth. In order to achieve long-term strategic goals, the accumulated R&D capabilities will allow a company to develop innovations independently. Therefore, companies should not primarily rely on an imitation strategy but should put more emphasis on developing firm-internal R&D competencies and gain a leading position in the market.

The sustainable growth of an enterprise is closely related to achieving long term strategic goals. The realization of short-term strategic goals can lay the foundation for achieving long-term strategic goals in providing sufficient firm resources and more advanced technology, etc. But in order to achieve long-term strategic goals, managers have to decide whether the enterprise can move toward a higher level of R&D development. As a result, managers need to pay greater attention to these two types of strategic goals at the same time. It is worth noting that, according to our research results, although an imitation strategy is important for improvements of both the long-term and the short-term, the degree of achieving these goals is different. Compared with long-term performance, the improvement over the short-term seems to be more dependent on an imitation strategy. This result demonstrates that the enterprises need to constantly adjust their imitation strategy over time. Although in the early stages of development, an imitation strategy can generate a competitive advantage for companies due to faster market entry and cheaper prices. However, with the continuous growth of enterprises and the gradual growth of markets toward maturity, the importance of independent innovation capabilities of enterprises is becoming increasingly vital with enterprises hardly being able to rely entirely on an imitation strategy (Zhan et al., 2020). As discussed by Li and Kozhikode (2008), if enterprises actually lack the necessary absorptive capacity, they can only blindly imitate. But when an enterprise accumulated sufficient absorptive capacity to start with generating independent innovations, it might prefer to adjust the original imitation strategy and apply a creative imitation strategy by adding some innovative features to the existing solutions. Moreover, enterprises need to fully consider the industrial dynamics to implement the phased development strategies. With history-friendly simulation, the results of Malerba and Orsenigo (2002) show the intricate relations between micro-dynamics and macro-outcomes through the market structure evolution. Similarly, Yu et al. (2020) also found that there is an interaction between demand structure and technological regimes influencing, in turn, market dynamics. Therefore, companies should not only follow the generalized development pattern within the industry (Garavaglia, 2010) but also respond to changes in technology and the market environment in a timely manner.

### Implications

The research results of this article demonstrate that an imitation strategy can promote firm growth more strongly in non-OECD member countries under low-tech industry conditions and when the firm is striving to achieve just short-term strategic goals. Meanwhile, the effects of an imitation strategy on firm growth are slightly weaker in OECD member countries in high-tech industries and when it is aiming at attaining long-term strategic goals.

Taking these unique attributes into account, implementing an appropriate imitation strategy are essential for enterprises to generate sustained competitive advantages and to achieve sustainable long term development (Ali, 2021). In the literature, two forms of imitation have been discussed, namely, pure imitation and creative imitation. While a firm would directly replicate existing products of competitors when using pure imitation strategies, a firm would not only replicate but also improve or add new features on the original products of competitors when adopting creative imitation strategies (Lee and Zhou, 2012). Therefore, it seems sensible for enterprises to take these two forms of imitation into account when selecting appropriate imitation strategies, thereby promoting sustainable growth of the enterprise over the long term.

**FIGURE 3:**
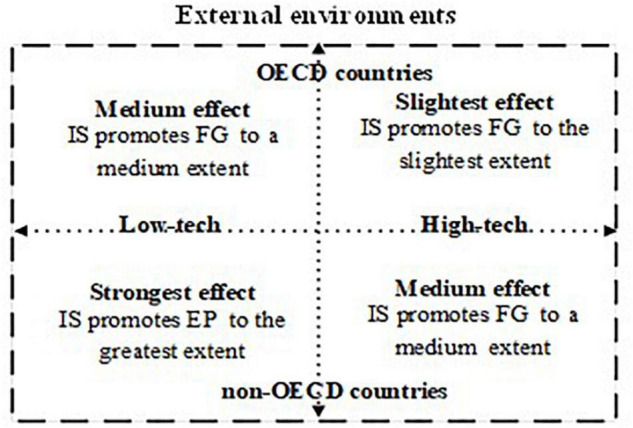
The distribution of the influence of imitation strategy on firm growth. “IS” represents “imitation strategy”; “FG” represents “firm growth.”

In order to display our results with respect to moderating effects of industry conditions and country-specific factors, a chart showing the influence of an imitation strategy on firm growth has been drawn (see Figure 3). It can be seen that in the non-OECD countries and under low-tech industry conditions, an imitation strategy will provide the strongest effects in promoting firm growth. In OECD countries and under high-tech industry conditions, enterprises should use, to a lesser extent, an imitation strategy and pay more attention to building their innovation capabilities to achieve sustainable development, because new innovative features on original products might provide a window of opportunity for them to enter markets. For the other two situations shown in Figure 3, an imitation strategy has just a moderate impact on firm growth.

**FIGURE 4:**
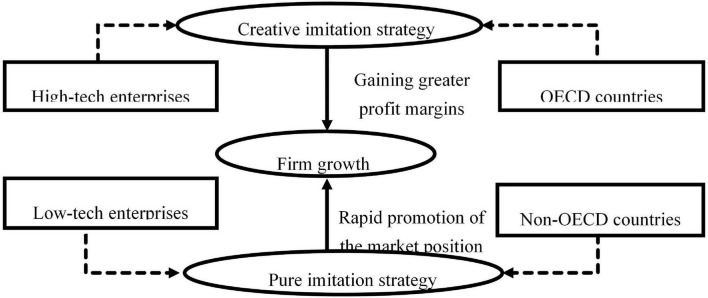
Specific selection of imitation strategy for firm growth based on the external environments of enterprises.

As the literature has shown that there are different strategies to adapt to various external conditions (Martin-Rios and Ciobanu, 2019), it will become meaningful for companies to choose a suitable imitation strategy that matches the conditions in their external environment. As shown in Figure 4, it is more beneficial for enterprises in a high-tech industry and in OECD Member countries to adopt a creative imitation strategy while it is more suitable for enterprises in a low-tech industry and in non-OECD countries to utilize a pure imitation strategy to obtain sustainable market advantage.

**FIGURE 5:**
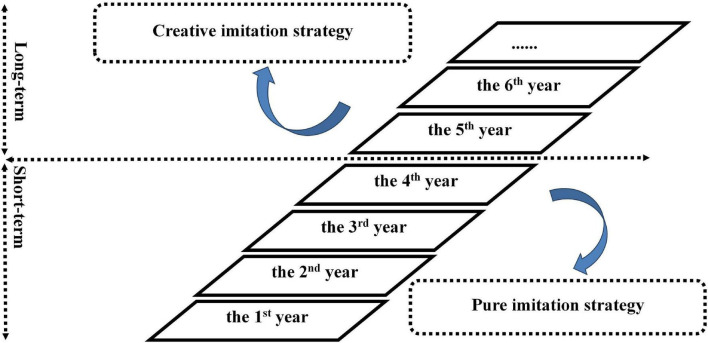
Specific selection of imitation strategy for sustainable competitive advantage.

Combining the meta-analysis results with the performance dimension (long versus short term), Figure 5 characterizes the appropriate timing for an imitation strategy. It can be seen that enterprises are better positioned in markets if they apply a pure imitation strategy to achieve short-term objectives. The imitation strategy enables enterprises to avoid risks, reduce costs, and catch up with competitors. While long-term objectives can be better achieved by utilizing a creative imitation strategy, aimed at modifying and optimizing the original products of competitors. New features or the updating functions of the products might be more appealing to customers when they are already satisfied with existing products in the market.

The findings of this paper are also of great significance to policymakers. Although the results of this study show that an imitation strategy is more effective in promoting firm growth under the low-tech industry conditions and when aiming at achieving short-term strategic goals in non-OECD countries, policymakers also need to pay attention to several factors that led to this result. It is hard to promote firm growth through R&D activities under low-tech industry conditions or in non-OECD countries due to the probable lack of R&D resources and the inadequacy of infrastructure construction. However, the ability of independent innovation may be an essential factor in ensuring the long-term development of an enterprise. Therefore, policymakers need to provide enterprises in the low-tech industry conditions or in non-OECD countries preferential policies for technology R&D to promote long-term firm growth.

## Conclusion

Although much attention has been paid in the academic literature to study the relationship between imitation strategy and firm growth over the past years, the studies in this area have been generated rather inconsistent results and more research is needed to deal with these shortcomings. To overcome the sampling errors and sample size limitations of single studies in this area, this paper adopted a meta-analysis method to find realistic and statistically proven evidence for the relationship between an imitation strategy and firm growth over the long term. In addition, this paper further examined the moderating effects of industry conditions, country-specific factors, and (short versus long-term) performance dimension. It further explored whether the inconsistent results of existing research studies were caused by moderating variables. In this context, the paper derived a conceptual framework from the literature to explain the relationship between an imitation strategy and firm growth. The research results show that, in non-OECD countries, under the low-tech industry conditions and when aiming at achieving short-term strategic goals, an imitation strategy is more effective in promoting firm growth. The research results of this paper can provide further insights for managers to choose an appropriate imitation strategy based on their choice between a pure or creative imitation strategy, thereby gaining a sustainable competitive advantage over the long term. Besides, the findings of this paper are also of great significance to policymakers. For enterprises in low-tech industries or non-OECD countries R&D, easy access to R&D incentives and support should be provided to enhance the innovation capabilities which could promote long-term firm growth.

## Data Availability Statement

The original contributions presented in the study are included in the article/supplementary material, further inquiries can be directed to the corresponding author.

## Author Contributions

HP designed and conceptualized the study. HP, CZ, and TS finished the literature search and data analysis. BS and CZ wrote and revised the manuscript. All authors read and approved the final manuscript.

## Conflict of Interest

The authors declare that the research was conducted in the absence of any commercial or financial relationships that could be construed as a potential conflict of interest.

## Publisher’s Note

All claims expressed in this article are solely those of the authors and do not necessarily represent those of their affiliated organizations, or those of the publisher, the editors and the reviewers. Any product that may be evaluated in this article, or claim that may be made by its manufacturer, is not guaranteed or endorsed by the publisher.
